# Prospective T1 mapping to assess gadolinium retention in brain after intrathecal gadobutrol

**DOI:** 10.1007/s00234-023-03198-7

**Published:** 2023-07-21

**Authors:** Geir Ringstad, Lars Magnus Valnes, Svein Are Sirirud Vatnehol, Are Hugo Pripp, Per Kristian Eide

**Affiliations:** 1 Department of Radiology, Oslo University Hospital- Rikshospitalet, Oslo, Norway; 2 Department of Geriatrics and Internal Medicine, Sorlandet Hospital, Arendal, Norway; 3 Department of Neurosurgery, Oslo University Hospital – Rikshospitalet, Postboks 4950 Nydalen, 0424 Oslo, Norway; 4 The Interventional Centre, Oslo University Hospital – Rikshospitalet, Oslo, Norway; 5 Institute of Optometry Radiography and Lighting Design, Faculty of Health and Social Sciences, University of South Eastern Norway, Drammen, Norway; 6 Oslo Centre of Biostatistics and Epidemiology, Research Support Services, Oslo University Hospital, Oslo, Norway; 7 Faculty of Health Sciences, Oslo Metropolitan University, Oslo, Norway; 8 Institute of Clinical Medicine, Faculty of Medicine, University of Oslo, Oslo, Norway

**Keywords:** Gadolinium-based contrast agents, Gadobutrol, Intrathecal, Cerebrospinal fluid, Magnetic resonance imaging, Retention

## Abstract

**Purpose:**

A possible pathway behind gadolinium retention in brain is leakage of contrast agents from blood to cerebrospinal fluid and entry into brain along perivascular (glymphatic) pathways. The object of this study was to assess for signs of gadolinium retention in brain 4 weeks after intrathecal contrast enhanced MRI.

**Methods:**

We prospectively applied standardized T1 mapping of the brain before and 4 weeks after intrathecal administration of 0.5 mmol gadobutrol in patients under work-up of cerebrospinal fluid circulation disorders. Due to methodological limitations, a safety margin for percentage change in T1 time was set to 3%. Region-wise differences were assessed by pairwise comparison using *t*-tests and forest plots, and statistical significance was accepted at .05 level (two-tailed).

**Results:**

In a cohort of 76 participants (mean age 47.2 years **±** 17.9 [standard deviation], 47 women), T1 relaxation times remained unchanged in cerebral cortex and basal ganglia 4 weeks after intrathecal gadobutrol. T1 was reduced from 1082 **±** 46.7 ms to 1070.6 **±** 36.5 ms (0.98 **±**2.9%) (mean [standard deviation]) (*p*=0.001) in white matter, thus within the pre-defined 3% safety margin. The brain stem and cerebellum could not be assessed due to poor alignment of posterior fossa structures at scans from different time points.

**Conclusion:**

Gadolinium retention was not detected in the cerebral hemispheres 4 weeks after an intrathecal dose of 0.5 mmol gadobutrol, implying that presence of contrast agents in cerebrospinal fluid is of minor importance for gadolinium retention in brain.

## Introduction

Mounting evidence has shown gadolinium to be retained in the human brain after intravenous administration of gadolinium-based contrast agents (GBCAs) [1]. Although the largest concentrations of gadolinium have been measured in the dentate nucleus and globus pallidus, inductively coupled plasma mass spectrometry has confirmed widespread low-level deposits of gadolinium in the entire human brain after multiple [2–4], and even single [5], GBCA exposures.

Possible pathways for GBCAs into brain tissue are from blood through breakages in the BBB and via the much leakier blood-cerebrospinal fluid (CSF) barrier. Gadolinium deposition in the brain has been demonstrated in the absence of BBB damage [6], suggesting that entry via CSF may play an important role. In fact, all marketed GBCAs, regardless of class, leak almost instantaneously from blood into rodent CSF [7, 8] and are found to enter human CSF both at MRI [9] and in CSF samples [10], being detectable in CSF for weeks [11]. Entry of GBCA from CSF to brain tissue was therefore hypothesized to occur from surface along perivascular spaces surrounding arteries [12], a main element of the glymphatic pathway [13]. Indeed, human studies have confirmed brain-wide enhancement 24–48 h after administration of GBCA directly into CSF (intrathecal), where the highest degrees of enrichment occurred in cerebral cortex, and particularly in regions adjacent to the large artery trunks at the brain surface [14]. T1 shortening in cerebral cortex has been shown to persist for at least 2–3 days after an intrathecal dose of 0.5 mmol [15].

Many discrepancies between MRI studies of gadolinium retention may be related to the studies’ retrospective design, small sample size, and heterogeneity with use of different patient groups, scanners, field strengths, or imaging protocols [1]. For instance, pulse sequence may directly affect T1 hyperintensity in brain tissue and affect comparisons of ratios at spin echo and gradient echo sequences, which provide different intrinsic gray-white matter contrast [16], and should not be used interchangeably. On the other hand, quantitative mapping of T1 relaxation time represents our most sensitive tool to detect and quantitatively assess subtle signs of T1 shortening from the presence of gadolinium in brain tissue. Estimations of T1 relaxation times have previously been applied in cross-sectional studies of gadolinium retention in brain [17–20], but not prospectively before and after administration of a contrast agent.

Here, we explored the hypothesis that gadolinium is retained in the brain after entry from CSF. The aim of this study was therefore to utilize prospective T1 mapping of the brain to assess for signs of gadolinium retention following intrathecal administration of a macrocyclic GBCA.

## Materials and methods

### Approvals and study design

The study was approved by The Regional Committee for Medical and Health Research Ethics (REK) of Health Region South-East, Norway (2015/96), The Institutional Review Board of Oslo university hospital (2015/1868) and The National Medicines Agency (15/04932-7), and included participants after written and oral informed consent. The study was conducted according to ethical standards of the Helsinki Declaration of 1975 (and as revised in 1983).

We applied a prospective and observational study design, comparing T1 time before and 4 weeks after intrathecal injection of gadobutrol.

### Participants

The patient cohort (Table 1) included consecutive individuals who underwent intrathecal contrast-enhanced MRI as part of their neurosurgical work-up for suspected CSF circulation disorders within the Department of Neurosurgery at Oslo University Hospital-Rikshospitalet, Norway, and who were imaged with T1 mapping during a study period lasting from October 2015 to November 2019.

**Table 1:** Demographic and clinical information about the study group

	Total material
*N*	76
*Sex (F/M)*	47/29
*Age (years)*	47.2 ± 17.9
*BMI (kg/m*^*2*^*)*	27.7 ± 5.0
*Clinical diagnosis prior to MRI*	
Normal pressure hydrocephalus	17 (22.1%)
Spontaneous intracranial hypotension	11 (14.3%)
Idiopathic intracranial hypertension	9 (11.7%)
Pineal cyst	15 (19.5%)
Arachnoid cyst (non-surgery)	17 (22.1%)
Hydrocephalus conditions	7 (9.1%)
*GFR (ml/min/1.73 m*^*2*^*)*	92.2 ± 19.3

*BMI* body mass index, *GFR* glomerular filtration rate. Categorical data presented as numbers; continuous data presented as mean ± standard deviation

T1 maps were obtained immediately before (referred to as *Pre*) intrathecal injection of 0.5 ml of 1.0 mmol/ml gadobutrol (Gadovist™ [EU]; Gadavist® [USA], Bayer) at the lumbar level, and repeated after 4 weeks (referred to as *4 weeks*). Pre and 4 weeks scans were obtained at similar times of the day (all before noon). For anatomical co-registration, we also obtained a 3D T1-weighted volume acquisition in parallel with T1 mapping. The lumbar injection puncture was performed as previously described [14].

None of the participants received any intravenous GBCA injection during the same scan or during the observation period of 4 weeks.

### Magnetic resonance imaging

All participants were imaged at both time points (Pre and 4 weeks) in the same 3 Tesla Philips Ingenia MRI scanner (Philips Medical Systems) with equal imaging protocol settings to quantify T1 time using a 3D Inversion Recovery Look-Locker turbo field echo planar imaging [21]. Main imaging parameters were as follows: repetition time = “shortest” (typically 36 ms), echo time = “shortest” (typically 17 ms), minimum inversion time = “shortest” (typically 19.6 ms), phase interval = 400.3ms, no. phases = 12, total cycle duration = 4815 ms, field of view = 245 × 211 cm, acquisition matrix = 1.97 × 3.44 × 4mm, reconstruction matrix = 1.39 × 1.39 mm^2^, scan time = 2 min and 39 s. Imaging parameters for the 3D T1 gradient echo volumetric scan were as follows: Repetition time = “shortest” (typically 5.1 ms), echo time = “shortest” (typically 2.3 ms), flip angle = 8°, field of view = 256 × 256 cm^2^ and matrix 256 × 256 pixels (reconstructed to 512 × 512) with total scan time 6 min and 29 s.

### Computation of T1 times and co-registration

Computation of T1 times and co-registration of images was performed by LMV, who was blinded to clinical diagnoses and other patient data given in Table 1. T1 times was computed by using polarity recovery of the magnetization, curve fitting with three parameters using Levenberg-Marquardt algorithm [22] and Look-Locker correction with the excitation angle in the curve fitting [23].

FreeSurfer (version 6.0) (http://surfer.nmr.mgh.harvard.edu/) was used to obtain segmentation of the 3D T1-weighted volume acquisitions.

**Fig. 1:**
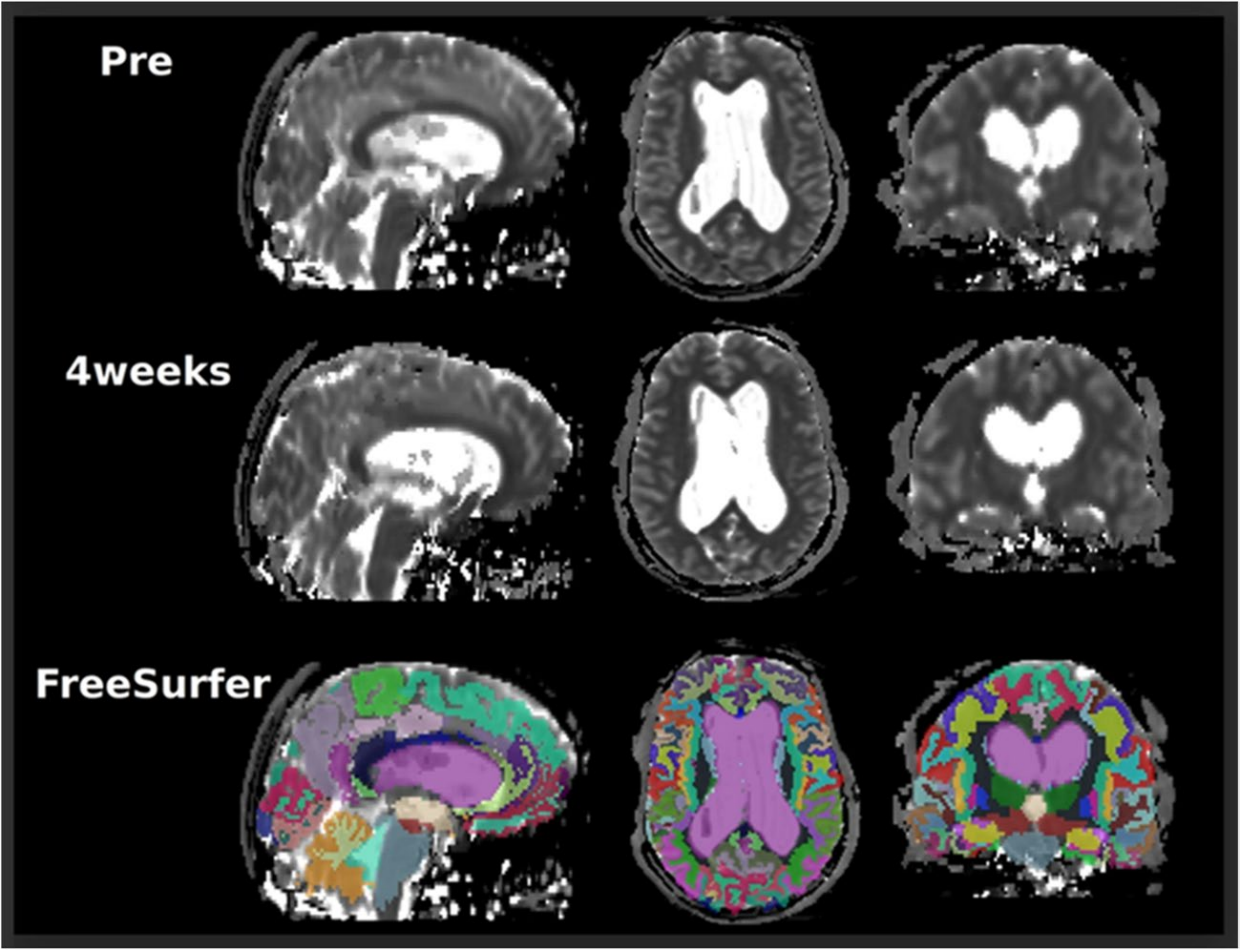
T1 maps derived from 3D Inversion Recovery Look-Locker turbo field echo planar imaging before (upper row) and 4 weeks (middle row) after intrathecal injection of 0.5 mmol gadobutrol. The prospective study design allowed for identical MRI scanner settings in all patients at both time points. FreeSurfer (version 6.0) (http://surfer.nmr.mgh.harvard.edu/) was used to obtain segmentation of 3D T1-weighted volume acquisitions and down-sampled onto the T1 map resolution (lower row)

T1 times were aligned with the segmentations by using the T1 times to create a synthetic T1 weighted MR image. Then, each of the synthetic MR images were co-registered with the corresponding 3D T1 weighted volume acquisition in FreeSurfer, and the resulting transformation was applied to the T1 times (Fig. 1).

The reliability of T1 measurements has previously been estimated to 5.0% for inversion recovery Look-Locker echo planar imaging across phantoms with a T1 range of 650–1900 ms [21]. There is sparse literature investigating the in vivo repeatability of T1 quantification in brain at 3T, repeatability from a previous human study of the MR fingerprinting technique defined a 3% change in T1 time as a reasonable safety margin [24]. Based on this, we defined a conservative safety margin of 3% change in T1 time between Pre and 4 weeks, i.e., any change in T1 time within 3% was assumed inherent with the T1 mapping methodology itself and not conclusive of gadolinium retention.

Volumetric changes between Pre and 4 weeks were estimated for the same regions as we assessed for change in T1 times. As Pre and 4 weeks scans were obtained before noon, we did not expect diurnal dependent changes in brain volume. However, as the FreeSurfer regions are down-sampled onto the T1 map resolution, we expect that there will be volume changes due to fact that a voxel can only have one label. Hence, volumetric changes could therefore be considered mainly a byproduct of the co-registration and a measure of its accuracy.

### Statistical analyses

SPSS version 27 (IBM Corporation, Armonk, NY) and Stata/SE 16.1 (StataCrop LLC, College Station, TX) were used for statistical analyses.

Artifacts, like aliasing, can cause the computed T1 times to be unrealistic, i.e., negative and exceeding 100,000 ms. Therefore, we limited the T1 times to the interval from 0 to 10000 ms, which spans a range significantly larger than reported T1 times in gray and white matter.

Continuous data were presented as mean (standard deviation) or mean (95% confidence intervals), as appropriate. Differences in T1 time before and after intrathecal gadobutrol were determined by paired *t*-test or one-sample *t*-test of percentage change and described by forest plots.

Associations between age and T1 time, and between age and change in T1 time, were assessed with Pearson correlation analysis. Plots and graphical methods evaluated normality of the data.

Statistical significance was accepted at the .05 level (two-tailed).

## Results

### Participants

MRI was obtained in 88 participants between October 2015 and November 2019. Twelve participants were excluded due to registration- and segmentation errors at Pre- or 4 weeks Look-Locker acquisitions, leaving a cohort of 76 patients. Demographic data and tentative diagnoses of the included participants are presented in Table 1.

### Alignment of segmentations

The Look-Locker acquisitions had a tendency to curve the posterior fossa structures slightly; therefore, T1 times did not align well with the segmentation in this region. Additionally, the posterior fossa structures were for some subjects not fully covered by the image acquisition. The brain stem and cerebellum therefore had to be excluded from the analysis.

### T1 relaxation times

**Fig. 2:**
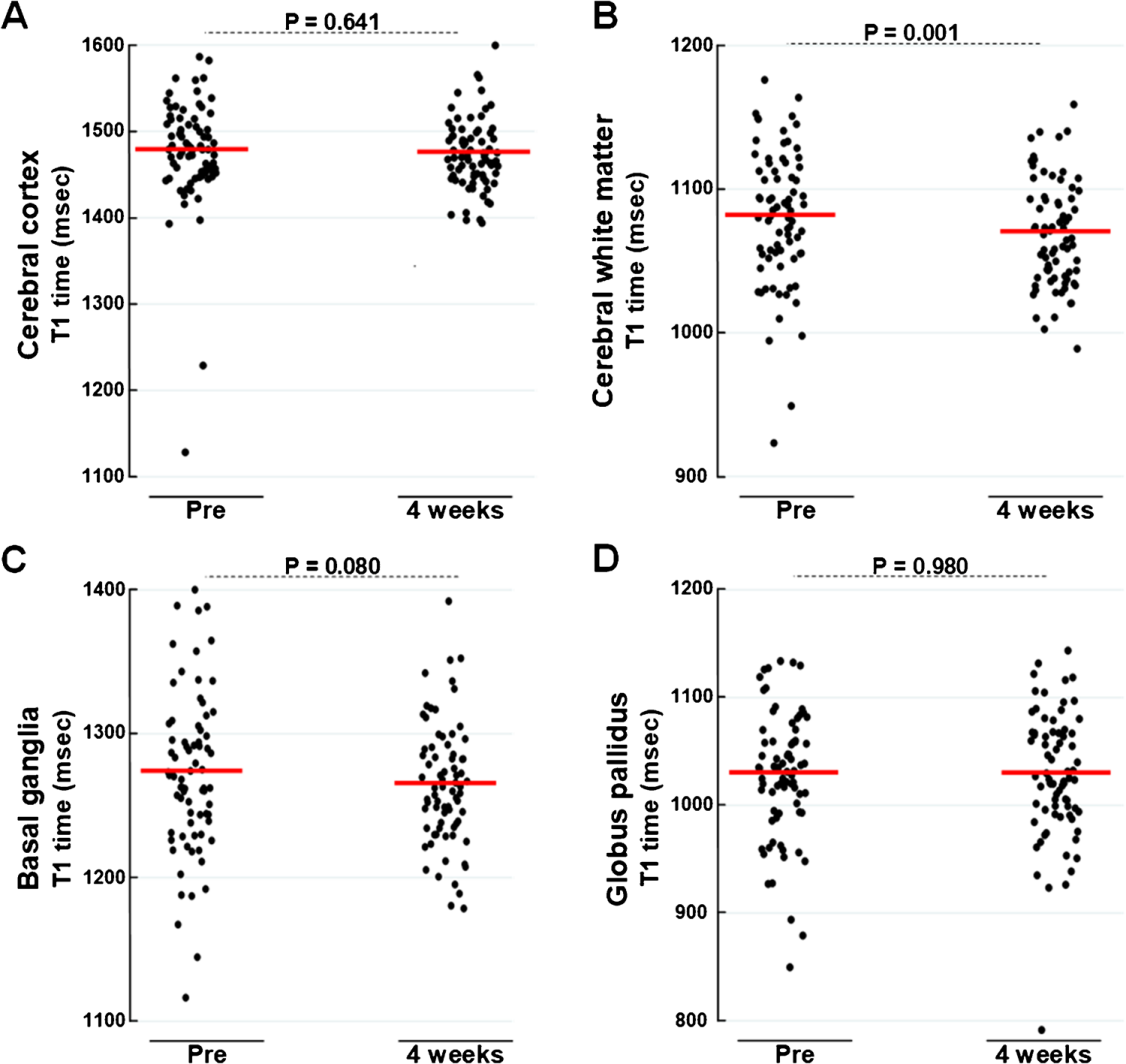
The T1 time (ms) before (Pre) and 4 weeks after intrathecal gadobutrol (0.5 mmol) within **A** cerebral cortex, **B** subcortical white matter, **C** basal ganglia, and **D** globus pallidus. Data presented as mean (red line) and individual measures indicated as dots. Significance levels are indicated (*t*-test)

T1 relaxation times at baseline and at 4 weeks are given for different sub-regions within the cerebral hemispheres in Table 2. Distribution of T1 times at Pre and 4 weeks in cerebral cortex, cerebral white matter, and basal ganglia, including the globus pallidus, is shown as scatter plot in Fig. 2.

**Table 2:** Differences in T1 relaxation time (msec) before (Pre) and 4 weeks after intrathecal gadobutrol (0.5 mmol) within some brain regions

Anatomical region	T1 time (msec) before (Pre) and after 4 weeks							Percentage change in T1 time from Pre to 4 weeks			
	Pre			After 4 weeks				Percentage change			
	Mean	±	SD	Mean	±	SD	^a^*P*-value	Mean	±	SD	^b^*P*-value
Main regions											
*Cerebral cortex*	1479.3	**±**	61.2	1476.4	**±**	33.8	0.641	−0.05	**±**	4.6	0.932
*Subcortical white matter*	1082.1	**±**	46.7	1070.6	**±**	36.5	0.001	−0.98	**±**	2.9	0.004
*Basal ganglia*	1274.3	**±**	55.0	1265.7	**±**	41.2	0.080	−0.58	**±**	3.3	0.137
Other regions											
*Frontal gray matter*	1493.1	**±**	67.5	1489.6	**±**	41.5	0.625	−0.05	**±**	5.0	0.927
*Frontal white matter*	1085.2	**±**	50.2	1075.9	**±**	41.6	0.013	−0.78	**±**	3.1	0.030
*Temporal gray matter*	1478.7	**±**	63.4	1473.8	**±**	38.6	0.464	−0.17	**±**	4.7	0.751
*Temporal white matter*	1115.4	±	55.3	1098.6	±	47.6	0.002	−1.39	±	4.2	0.005
*Parietal gray matter*	1485.8	**±**	66.1	1484.0	**±**	42.0	0.788	0.04	**±**	4.8	0.939
*Parietal white matter*	1032.2	**±**	51.2	1020.3	**±**	39.5	0.002	−1.05	**±**	3.3	0.006
*Occipital gray matter*	1417.3	**±**	67.6	1417.7	**±**	47.2	0.958	0.18	**±**	4.5	0.733
*Occipital white matter*	1104.3	**±**	59.9	1096.1	**±**	50.2	0.115	−0.61	**±**	4.1	0.198
*Insular cortex*	1555.5	**±**	81.6	1546.8	**±**	60.4	0.375	−0.31	**±**	6.3	0.673
*Subinsular white matter*	1037.3	**±**	47.1	1033.7	**±**	36.2	0.394	−0.24	**±**	3.6	0.569
*Pallidum*	1030.6	**±**	60.6	1030.4	**±**	59.1	0.980	−0.22	**±**	6.9	0.781

Data presented as mean ± standard deviation for total cohort (*n*=76). ^a^*P*-value (4 weeks–Pre; *t*-test). ^b^*P*-value (percentage change; *t*-test). *GM* gray matter, *WM* white matter, *Ns* non-significant differences between groups

**Fig. 3:**
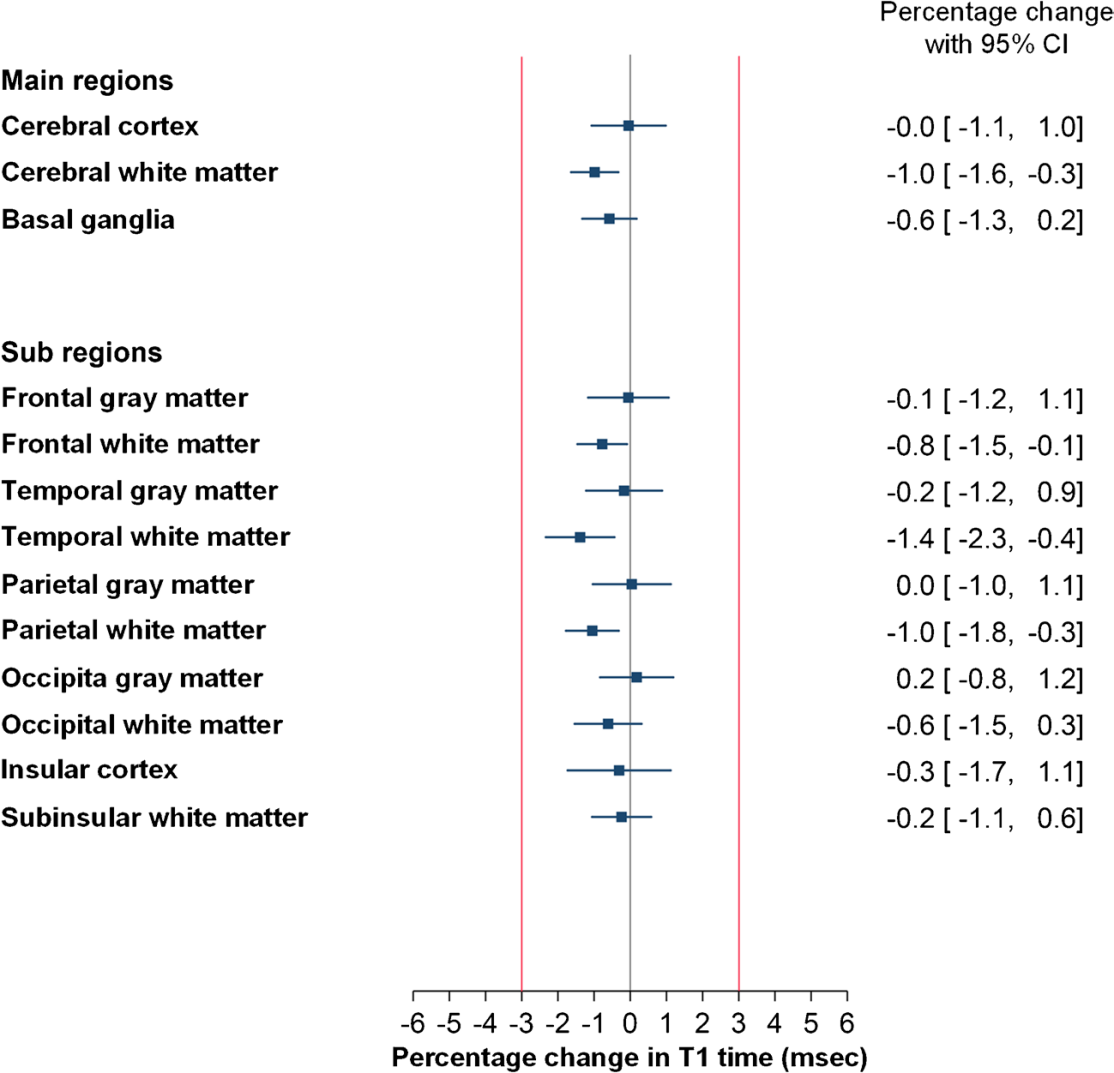
Forest plot of percentage change in T1 time between Pre and 4 weeks for the brain regions included in the analysis. Presence of gadolinium in brain tissue after 4 weeks is expected to shorten the T1 time. The red lines indicate a pre-defined 3% safety margin for change in T1 time, i.e. any change within **±**3% was considered to be within limitations inherent with the methodology. To the right for each plot is presented percentage change with 95% confidence intervals

T1 time was unchanged at 4 weeks in cerebral cortex and basal ganglia, including the globus pallidus. In cerebral white matter, T1 relaxation time was reduced with mean (SD) 0.98 (**±**2.9)% (*p*=0.004), which was within the pre-defined 3% safety margin for inherent methodological limitations (Fig. 3).

### Brain volume change between Pre and 4 weeks

Brain volumes at Pre and 4 weeks, and percentage change in brain volume at 4 weeks, are given for all main regions and sub-regions (brain lobes) in Table 3.

**Table 3:** Difference in brain volume before (Pre) and 4 weeks after intrathecal gadobutrol (0.5 mmol) within some brain regions

Anatomical region	Brain volume (ml) before (Pre) and after 4 weeks							Percentage change in brain volume from Pre to 4 weeks			
	Pre			After 4 weeks				Percentage change			
	Mean	±	SD	Mean	±	SD	^a^*P*-value	Mean	±	SD	^b^*P*-value
Main regions											
*Cerebral cortex*	500.6	**±**	45.4	491.5	**±**	54.6	0.006	−1.86	**±**	5.6	0.005
*Subcortical white matter*	333.3	**±**	40.5	333.6	**±**	40.7	0.431	0.08	**±**	0.9	0.440
*Basal ganglia*	22.8	**±**	2.4	22.8	**±**	2.5	0.677	−0.13	**±**	2.4	0.637
Other regions											
*Frontal gray matter*	198.0	**±**	20.6	195.3	**±**	24.0	0.043	−1.40	**±**	5.9	0.041
*Frontal white matter*	146.6	**±**	19.3	146.8	**±**	19.3	0.224	0.17	**±**	1.1	0.185
*Temporal gray matter*	106.7	**±**	10.8	103.9	**±**	12.7	<0.001	−2.70	**±**	5.9	<0.001
*Temporal white matter*	53.1	±	7.3	53.1	±	7.4	0.778	−0.07	±	1.6	0.703
*Parietal gray matter*	139.6	**±**	12.9	137.6	**±**	14.9	0.032	−1.39	**±**	5.6	0.034
*Parietal white matter*	99.4	**±**	12.0	99.3	**±**	12.0	0.747	−0.04	**±**	1.2	0.784
*Occipital gray matter*	56.4	**±**	6.4	54.7	**±**	7.1	<0.001	−2.97	**±**	5.6	<0.001
*Occipital white matter*	34.3	**±**	5.3	34.4	**±**	5.4	0.237	0.38	**±**	2.7	0.216
*Insular cortex*	15.4	**±**	1.6	15.3	**±**	1.9	0.405	−0.63	**±**	6.8	0.419
*Subinsular white matter*	19.4	**±**	2.1	19.6	**±**	2.1	0.072	0.87	**±**	3.7	0.046
*Pallidum*	4.0	**±**	0.5	4.0	**±**	0.5	0.874	0.21	**±**	6.7	0.789

Data presented as mean ± standard deviation for total cohort (*n*=76). ^a^*P*-value (4 weeks–Pre; *t*-test). ^b^*P*-value (percentage change; paired test). *GM* gray matter, *WM* white matter, *Ns* non-significant differences between groups

Percentage change in overall cerebral cortex volume from Pre to 4 weeks was mean (SD) −1.86 ± 5.6 (*p*=0.005), indicating some degree of misfit at co-registration, but to a minor degree. Volumetric change occurred also for some sub-regions including frontal cortex (*p*=0.041), temporal cortex (*p*<0.001), parietal cortex (*p*=0.034), occipital cortex (*p*<0.001), and sub-insula white matter (*p*=0.046), but with modest effect size (range −2.97 to 0.87%). There were no volumetric changes for cerebral white matter or basal ganglia.

### Associations between participant age and T1 times

**Fig. 4:**
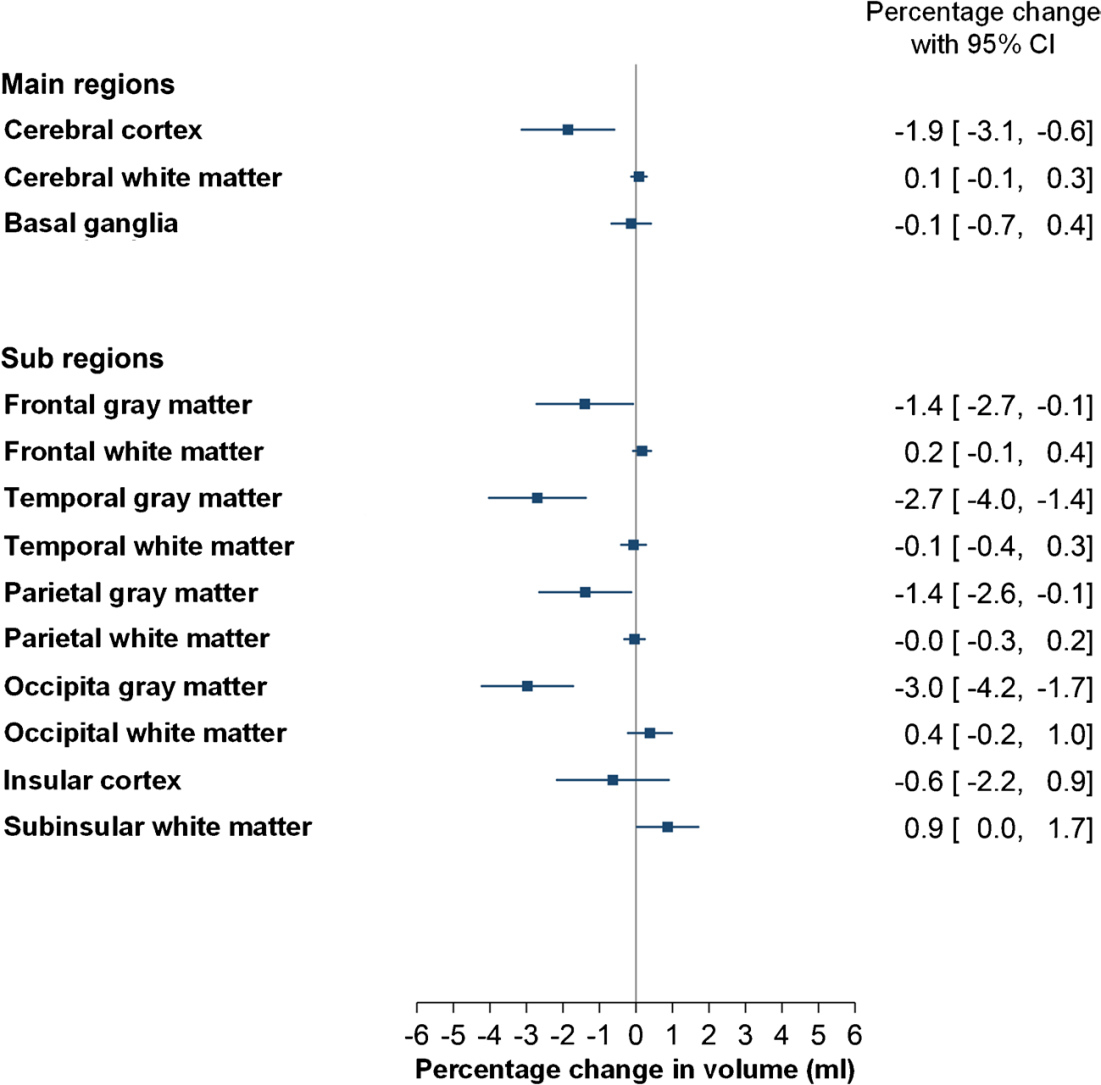
Forest plot of percentage change in volume of brain regions included in analysis. To the right for each plot is presented percentage change with 95% confidence intervals (CI)

There were no correlations between age and T1 time in cerebral main regions or sub-regions at Pre and at 4 weeks (Table 4, Fig. 4). Neither were there any correlations between age and percentage change in T1 time at 4 weeks, indicating results were not affected by participant age.

**Table 4:** Correlations between age and T1 time (ms), and between age and change in T1 time (ms) 4 weeks after intrathecal gadobutrol (0.5 mmol) within some brain regions

Anatomical region	Correlations between T1 time (msec) and age				Correlations between percentage change in T1 time from Pre to 4 weeks	
	Pre		After 4 weeks		Percentage change	
	Correlation coefficients	*P*-value	Correlation coefficients	*P*-value	Correlation coefficients	*P*-value
Main regions						
*Cerebral cortex*	0.094	0.417	0.160	0.168	0.003	0.983
*Subcortical white matter*	−0.008	0.947	−0.002	0.984	0.01	0.957
*Basal ganglia*	0.053	0.647	−0.102	0.380	−0.17	0.147
Other regions						
*Frontal gray matter*	0.130	0.263	0.214	0.063	0.01	0.926
*Frontal white matter*	0.112	0.334	0.137	0.239	0.005	0.963
*Temporal gray matter*	0.012	0.918	−0.066	0.572	−0.04	0.707
*Temporal white matter*	−0.153	0.187	−0.178	0.124	−0.01	0.947
*Parietal parietal matter*	0.049	0.674	0.165	0.155	0.06	0.608
*Parietal white matter*	−0.019	0.869	0.069	0.554	0.11	0.365
*Occipital gray matter*	0.146	0.208	0.116	0.320	−0.07	0.564
*Occipital white matter*	−0.082	0.480	−0.201	0.081	−0.12	0.309
*Insular cortex*	0.089	0.444	0.163	0.158	0.02	0.847
*Subinsular white matter*	0.049	0.675	0.108	0.351	0.04	0.732
*Pallidum*	0.008	0.946	−0.005	0.963	−0.01	0.914

Data presented as Pearson correlation coefficients with *P*-values for total cohort (*n*=76)

## Discussion

In this prospective study, we utilized T1 mapping of the brain to assess for signs of gadolinium retention 4 weeks after intrathecal administration of the macrocyclic GBCA gadobutrol. No conclusive signs of retention in the cerebral hemispheres were found, whereas posterior fossa structures had to be excluded from the analysis due to misfit of co-registrations.

Considering technical limitations inherent with repeated T1 relaxometry, we defined any changes in T1 time within 3% to fall within limits of measurement error. In a broader context, the 0.98% T1 decrease in cerebral white matter is for instance far less than that reported from GBCA leakage into the same region 30 min after a regular intravenous dose of 0.1 mmol/kg [25]. Furthermore, from our previous experiences with intrathecal MRI, enhancement of GBCA in brain occurs from the surface in a centripetal fashion, and is therefore by far highest within the cerebral cortex and much more subtle in the underlying white matter [14]. To this end, the region previously shown to have the highest contrast load 24 h after intrathecal injection, the cerebral cortex, showed at 4 weeks no sign of change in T1 time, nor in the basal ganglia, where peak enhancement after intrathecal administration is typically very limited. While signs of gadolinium retention in the basal ganglia have been shown at MRI after intrathecal administration of linear GBCA in a limited number of patients [26, 27], the current dataset corroborates other, larger studies where no signs of retention in brain could be detected at T1-weighted imaging after intrathecal macrocyclic GBCA [14, 28, 29].

A variety of techniques has been developed for quantitative T_1_ mapping. The choice of technique represents in general a compromise between accuracy, precision, and imaging time. T1 mapping protocols are known to produce stable T1 values in phantoms, but not in vivo, where variability of T1 relaxation times in the order of 30% has been reported in brain tissue, which is attributed mainly to different scanners and protocols [30]. A large variation of reported T1 values thus exists in literature for the same tissues and field strengths, and the T1 relaxation times we found are at the high end of those reported previously. A previous study using higher in-plane image resolution (1 mm) than here (1.39 × 1.39 mm) reported that standard deviation across Look-Locker based measurements was 19 ms in white matter and 33 ms in gray matter, corresponding to an accuracy of 3.5% and 3.2%, respectively [31]. In our current study, standard deviations of T1 times pre contrast and at 4 weeks were substantially higher (Table 2). One reason for this may be our lower image resolution, where partial volume effects should be expected to have influenced on precision of segmentations and thus on our region-specific measurements.

No GBCA has yet been approved for intrathecal use. Controlled studies of off-label applications are a mainstay of clinical advances, where benefit-to-risk ratio of intrathecal GBCA administration must always be taken into consideration. Intrathecal use of GBCAs is sporadic, but probably quite widespread, as one literature search from 2020 yielded 475 studies [32]. Concerns about intrathecal use relate to potential effects from immediate neurotoxicity and long-term deposition in brain [26]. A meta-analysis of 1036 patients who received intrathecal GBCA showed minor adverse events with dose 1 mmol or less, whereas all serious adverse events had occurred after a dose of 2 mmol or more (range, 2–10 mmol) [32]. More recent prospective studies of quite large patient cohorts for up to 12 months did not demonstrate signs of neurotoxicity with intrathecal gadobutrol in doses of 0.25–0.50 mmol and also showed that side effects were non-serious, temporary, and occurred with prevalence comparable to symptoms reported after spinal punctures [33, 34].

After the intravenous administration of gadobutrol in dose 0.1 mmol/kg, the total body dose of gadolinium is 16 times larger in an 80 kg subject compared to an intrathecal dose of 0.50 mmol, having an impact for risk of gadolinium retention in body tissues. In plasma, an intravenous dose of 0.1 mmol/kg peaks at a concentration of 0.59 mM [35], whereas an intrathecal dose of 0.5 mmol provides a peak concentration in blood of merely 0.0014 mM [36]. Furthermore, due to leakage of intravenous GBCA, concentration of gadolinium in the CSF can be up to 0.2 mM [37], while a patient dose of 0.5 mmol injected intrathecally yielded a concentration of 0.5 mM in selected regions of CSF [15] and 0.1 mM in the cerebral cortex [15]. An estimate of the concentration of the contrast agent entering into brain tissue after intravenous administration can be obtained when the concentration-time curve in the blood and the leakage coefficient of the cerebral vasculature are known. Subsequently, these input parameters can be used to model the transfer of contrast agent between the intra- and extravascular space based upon the concentration difference between these two compartments. When assuming the short-term evolution of contrast concentration in blood after intravenous injection based upon earlier work on the arterial input function in contrast agent based perfusion MRI methods [38, 39], the long-term evolution based on the works of Weinmann and Tofts [40, 41], and the leakage coefficient from measurements in the hippocampus of elderly subjects [42], one can estimate an approximate peak concentration of 0.09 mM in brain tissue from a standard intravenous dose of 0.1 mm/kg. This number should be considered a ballpark estimate, highly dependent on the assumed parameters. This concentration is comparable with estimated peak concentration in brain tissue after intrathecal administration (0.1 mM) [15]. The load of GBCA to the brain extra-vascular compartment may therefore be within the same range for intravenous and intrathecal administrations, or even higher for intravenous GBCAs in doses given at the upper limit of what is approved (0.3 mmol/kg). Concentrations of gadolinium in blood, brain, and CSF after intravenous and intrathecal administration in clinically relevant doses are given in Table 5.

**Table 5:** Comparison of intravenous (IV) and intrathecal (IT) gadobutrol in clinically relevant doses

	IV	IT
Blood Gd concentration (peak)	**0.59** mM (plasma) [35] (at 2 min, in dose 0.1 mm/kg)	**0.0014** mM (blood) [36] (at ~10 h)
CSF Gd concentration (peak)	**0.2** mM [37] (in dose 0.1 mmol/kg IV)	**0.5** mM [15] (0.5 mmol IT)
Brain Gd concentration (peak)	**0.09** mM (in dose 0.10 mmol/kg)*	**0.1** mM [15]

*Gd* gadolinium

*Estimate based on standard IV dose of GdDTPA/dimeglumine (0.1 mmol/kg) and *K*_*i*_ value of 1 × 10^−3^ min^−1^

**Fig. 5:**
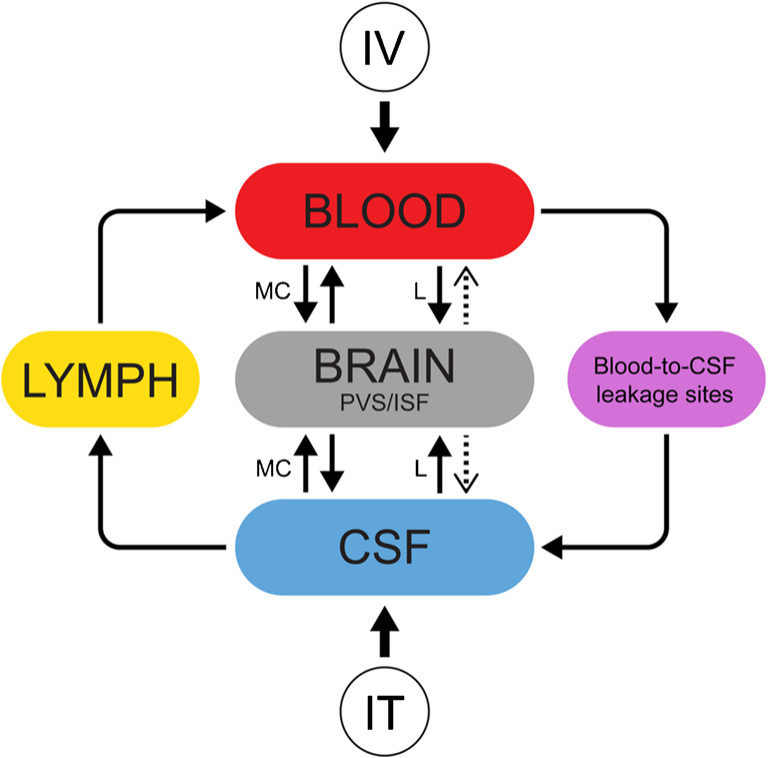
Schematic illustration of GBCA exchange between blood, cerebrospinal fluid (CSF), and brain perivascular space (PVS) and interstitial space (ISF) after intravenous (IV) and intrathecal (IT) administration, respectively. Whereas IT-administered contrast agents are mainly cleared from CSF to blood via lymphatic routes, GBCAs leak from blood to CSF along several leakage sites, including the choroid plexus, the ciliary body of the eye, along cortical veins, and cranial nerves. GBCA exchange with the brain tissue from both blood and CSF, where macrocyclic agents (MCs) have much lower risk of retention than linear agents (L)

Of note is the previous paper by Lee and co-workers [25], where a significant T1 shortening was observed to peak in white and particularly gray matter after 30 min. The authors interpreted this effect to be from GBCA leakage from blood to CSF and then entry into brain from surface. However, when GBCAs leak from blood to CSF, enhancement from into deeper parts of brain tissue should expected to take hours, not minutes. Previous studies with intrathecal enhanced MRI has shown enhancement (as evidence of CSF-ISF exchange) in brain to occur several hours after administration of contrast agent, particularly in deep white matter [14]. We therefore see it much more likely that the T1 shortening observed in the study by Lee and co-workers is due to leakage of contrast agent into brain tissue directly over the BBB, and not to represent CSF-ISF exchange within deep white matter. Subtle BBB damage with leakage of intravenous MRI contrast agents has previously been described in small-vessel disease, diabetes, and Alzheimer`s disease [43–46], but also in normal aging [42]. Since we found no signs of gadolinium retention in brain after an intrathecal contrast bolus in an amount that supersedes the amount leaking from blood into CSF, we may speculate that leakage over the BBB may be a more likely source of gadolinium retention in brain, rather than from leakage over the blood-CSF barrier. Increased risk of retention in brain with linear GBCAs likely applies to both pathways (Fig. 5).

## Limitations

It is scientifically not possible to prove equality, nor the absence of harm. Gadolinium retention may be below threshold of what can be detected at imaging [2], and it is possible that only MRI visible gadolinium species are detected [1]. Furthermore, T1 shortening effects of retained macrocyclic GBCAs may be weaker than T1 shortening from linear agents [47].

No phantom or human calibration studies to validate intra-scanner variability of T1 values were performed, which was beyond the scope of this clinical study. However, we defined a safety margin for T1 change in the low range (3%), which can be considered conservative, as reliability of T1 measurements with the methodology we applied has been reported to be 5% [21]. This safety margin, however, comes with a risk for a type 2 error, i.e., a false negative result.

Without coverage of posterior fossa structures, we cannot conclude about GBCA retention in the brain stem or the cerebellum. A further limitation is the possibility that our cycle time of 4815 ms is not long enough to avoid saturation of the longer T1 time components found in voxels consisting of a combination of normal white matter, CSF, and cortex tissue. A potential saturation of these longer T1 times could, in theory, overshadow a change in T1. Further technical improvements of the protocol in terms of cycle duration or resolution might remedy this limitation in later studies.

## Conclusion

In this prospective study of patients 4 weeks after receiving 0.5 mmol gadobutrol intrathecally, no conclusive signs of gadolinium retention in the cerebral hemispheres was detected by use of T1 mapping. The results suggests that presence of a macrocyclic contrast agent in cerebrospinal fluid is of minor importance to gadolinium retention in brain.
